# Characteristic Times for Gap Relaxation and Heat Escape in Nanothin NbTi Superconducting Filaments: Thickness Dependence and Effect of Substrate

**DOI:** 10.3390/nano14191585

**Published:** 2024-09-30

**Authors:** Khalil Harrabi, Abdelkrim Mekki, Milorad V. Milošević

**Affiliations:** 1 Physics Department, King Fahd University of Petroleum and Minerals, Dhahran 31261, Saudi Arabia; akmekki@kfupm.edu.sa; 2 Interdisciplinary Research Center (RC) for Intelligent Secure Systems, King Fahd University of Petroleum & Minerals (KFUPM), Dhahran 31261, Saudi Arabia; 3 Interdisciplinary Research Center (RC) for Advanced Material, King Fahd University of Petroleum & Minerals (KFUPM), Dhahran 31261, Saudi Arabia; 4 Department of Physics, University of Antwerp, Groenenborgerlaan 171, B-2020 Antwerp, Belgium

**Keywords:** critical current, hotspot, phase slip center, non-equilibrium superconductivity

## Abstract

We measured the temporal voltage response of NbTi superconducting filaments with varied nanoscale thicknesses to step current pulses that induce non-equilibrium superconducting states governed by a hot spot mechanism. Such detected voltage emerges after a delay time $t_{d}$, which is intimately connected to the gap relaxation and heat escape times. By employing time-dependent Ginzburg–Landau theory to link the delay time to the applied current, we determined that the gap relaxation time depends linearly on film thickness, aligning with the acoustic mismatch theory for phonon transmission at the superconductor–substrate interface. We thereby find a gap relaxation time of 104 ps per nm of thickness for NbTi films on polished sapphire. We further show that interfacial interaction with the substrate significantly impacts the gap relaxation time, with observed values of 9 ns on Si$O_{x}$, 6.8 ns on fused silica, and 5.2 ns on sapphire for a 50 nm thick NbTi strip at T=5.75 K. These insights are valuable for optimizing superconducting sensing technologies, particularly the single-photon detectors that operate in the transient regime of nanothin superconducting bridges and filaments.

## 1. Introduction

Both non-equilibrium superconductivity and thermal transport in superconducting materials have been of pertinent importance over the past decade for their direct relevance to established and emerging nano-electronic devices. For example, single-photon detectors based on superconducting nanowire meanders readily found their way to practical applications in optical communications and quantum technology [1]. Moreover, recent research showed that superconducting wires can function effectively as basic elements for detection even when structured at micrometer scales [2], rather than the traditionally used nanometer scales. This facilitates the fabrication of such detectors and increases the device area sensitive to photon impact, both advancing this technology.

It is well known that superconducting filaments can carry electrical current without dissipation as long as the current is kept below the critical value $I_{c}$, beyond which the non-equilibrium state is reached and the energy dissipates via formation of phase-slip centers and hot spots [3]. The formation of such resistive states results in the occurrence of well-defined resistance steps in the measured current–voltage (*I*-*V*) characteristics [4,5]. For example, corresponding voltage jumps were reported in the *I*-*V* characteristics of ultra-thin superconducting NbN nanowire grown on a sapphire substrate [6], where at low temperatures a crossover between the thermal and the quantum behavior in the phase-slip regimes was thoroughly studied. Quantum phase-slip centers were also investigated in a superconducting NbN wire of width reduced down to several superconducting coherence lengths (ξ) [7], and distinction has been made between the coherent and incoherent quantum phase-slip events, the latter being fostered by thermally activated fluctuations.

With regard to theory, the time-dependent Ginzburg–Landau (TDGL) theory is known to be the most effective formalism to capture the resistive state of current-carrying superconductors [3]. For example, this methodology was successfully used to describe the current-induced resistive state in 1D [8,9], 2D [10,11,12,13,14], and 3D [15,16,17,18,19] superconductors. This approach can also account for other sample-specific features such as pinning centers [20,21,22,23,24], disorder and spatial inhomogeneities [25], and external [18,26] and internal [27,28,29] magnetic fields, which all affect the dynamics of the superconducting state under current bias, and exhibited a very good agreement with most experiments to date [9,12,14,19,22,23,24,25].

On subject of technology, the creation of a localized non-equilibrium state (i.e., hot spot) under driving current is the working mechanism of the Superconducting Nanowire Single-Photon Detectors (SNSPDs) [30,31], hence the optimized thermal properties of these devices are essential for their reliable operation. In Ref. [32], such a hot spot was created in NbN waveguide-integrated superconducting nanowire under current bias and irradiation of photons, and the hot spot relaxation time was deduced. The heat dissipated in the localized hot spot is evacuated towards the substrate by phonons. The thermal boundary conductance between the superconducting film and the substrate quantifies the phonon rate emission and heat evacuation via the substrate. The quality of such thermal interfacing is a dominant feature that affects the performance and sensitivity of devices such as SNSPDs, as well as their jitter time, switching current, and efficiency [33]. The thermal boundary conductance between superconducting nanowires and different substrates were also measured using the hot spot current and the mismatch model [34]. These studies showed the importance of the substrate to the out-of-equilibrium dynamics of the superconducting state in current-carrying superconducting systems.

Therefore, in this work, we analyze the dependence of the dynamic properties of NbTi superconducting bridges under pulsed current on the thickness of the superconducting filament and the quality of its interface with the substrate, both having direct impact on the thermal mechanisms within any device made of such a filament. We have investigated a relatively novel NbTi material as an alternative to the conventional NbTiN or NbN, aiming to explore its potential advantages for detection electronics. We established that the delay time $t_{d}$ before transitioning to the resistive state decreases with increasing the current pulse amplitude. Using time-dependent Ginzburg–Landau theory, we then determined that the gap relaxation time depends linearly on film thickness, for a given substrate, which is consistent with the acoustic mismatch theory. We proceeded to investigate the non-equilibrium states in NbTi filaments on different substrates, finding that substrate material and the interface quality significantly impact gap relaxation and heat escape times of the superconducting nanostructure. These findings highlight the critical importance of film thickness and substrate choice for optimizing the superconducting electronics. Namely, the relaxation time, particularly the gap relaxation time, is a critical parameter in determining the suitability of superconducting materials for various applications where response dynamics is essential. Controlling this relaxation time is thus crucial for developing high-performance devices, including single-photon detectors, quantum computing systems, and other advanced superconducting circuits.

## 2. Experimental Setup and Detection of the Expected Dissipative States

**Figure 1:**
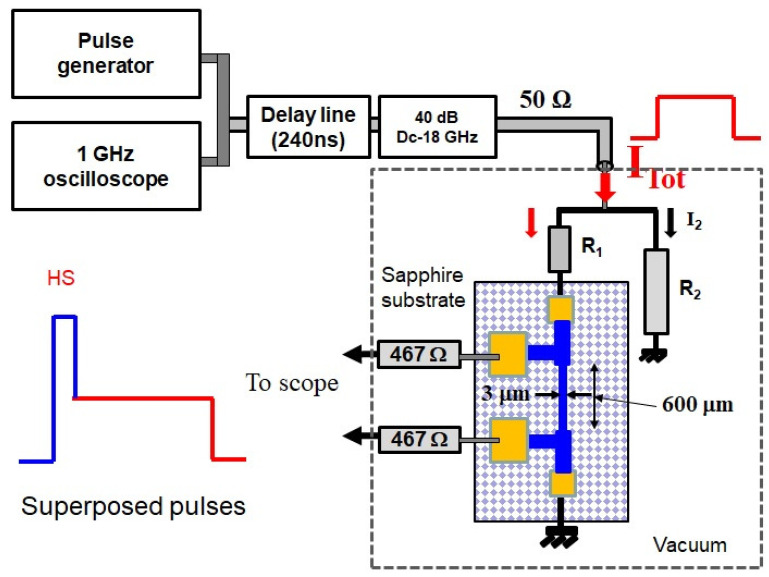
Schematics of the experiment: the lateral probes are connected to the oscilloscope through 467 Ω resistors to avoid any current deviation to the outside circuit. The sample is mounted in parallel to a resistor $R_{2}$ to ensure that constant current is fed through the sample. Two current pulses were superposed and sent to the filament: the shortest pulse is used to create a hot spot (HS), and the second one with the plateau serves to maintain it.

In this experiment, Niobium–Titanium (NbTi) wires with a width of 3.0 μm and varied thicknesses (30, 50, and 80 nm) were deposited on a sapphire substrate via sputtering in an Ar-N plasma under vacuum conditions (STAR-Cryoelectronics, Albuquerque, NM, USA). The superconducting wires and electrical connections were created using photolithography and ion milling, resulting in a sample layout featuring a 600 μm long wire for current pulse application, as shown in Figure 1. The sputtering deposition technique was chosen for fabrication because it has consistently proven to be both efficient and reliable for producing high-quality thin films. This method has been successfully applied in various advanced applications, such as single-photon detection and qubit fabrication [35], underscoring its relevance and effectiveness in ensuring the material’s performance and consistency. Voltage measurements were taken across the wire using a double-sided probe set. Additionally, 50 nm thick NbTi wires with a width of 5 μm were fabricated and deposited on sapphire, fused silica, and silicon oxide substrates. Current pulses were applied to the wire, which was grounded at the other end, with voltage measurements taken across the wire. The measurements were conducted under vacuum, using current pulses of variable amplitude, a 450 ns duration, and a 10 kHz repetition rate, applied via a 240 ns air-delay line. Voltage was monitored with a fast oscilloscope, and the samples exhibited superconducting transition temperatures around 8.0 K. In our investigation, we employed the pulse technique due to its significant advantage in controlling the growth of a hot spot once it is created, making it particularly useful in logic circuits. Unlike DC I-V characteristics, which often yield only a few observable steps and limit the ability to thoroughly study and control the superconducting or resisitive state, as noted by Tinkham [3], the pulse technique enables precise manipulation and analysis of hot spot dynamics, thereby enhancing the performance and reliability of superconducting logic circuits. This pulsed method can also be applied to single-photon detection and precise calibration of light sources, as shown in Ref. [36].

In a current transport measurement, the zero resistance survives up to a critical current $I_{c}$, beyond which dissipation arises through local onset of phase-slip lines [37,38]. Here, we defined the critical current as the minimum current that generates a detectable voltage with a delay time of around 440 ns. The phase-slips and/or vortex–antivortex pairs induced beyond $I_{c}$ exhibit high velocity [39] dependent on an applied magnetic field. The generated quasi-particles diffuse over an inelastic length, cause heating and dissipation, and a clear voltage signal emerges.

Within a phase-slip line (PSL), the collapse and restoration of the order parameter occurs at the Josephson frequency, and the phase of the order parameter changes by 2π across the PSL. In a current-biased transport measurement, the normal current within a PSL is the source of Joule heating, leading to hot spots and nucleation of local normal zones above a certain thermal threshold current $I_{h}$, dependent upon the cooling conditions. Depending on sample specifics, the two currents $I_{c}$(T) and $I_{h}$(T), have different dependencies, and the crossing temperature T* between these two currents defines the nucleation temperature of a PSL, which is usually confined to a restricted temperature range close to $T_{c}$. However, below T* a hot spot regime is found. Some thin films showed a completely different behavior where the PSL regime extends over the entire range of temperature below $T_{c}$ [40].

Therefore, a superconducting bridge biased with a current exceeding the switching value can present two different thermal behaviors. In these two states, the dissipated energy leads to attaining a temperature smaller or larger than $T_{c}$. Such dissipation was discussed in superconducting bridges near $T_{c}$, where the theoretical study and numerical calculations showed that in both thermal and quasi-equilibrium limits the delay time $t_{d}$ is independent of temperature, which contradicts with the non-thermal model that showed a strong temperature dependence [8,11]. Therefore in this work, we revisit the issue both experimentally and theoretically, and use the observed behavior of the delay time to extract the characteristic dissipative times for thin superconducting filaments on different substrates—as relevant for applications in superconducting electronics and sensing.

## 3. Creation of a Hot Spot Using an Electrical Current Pulse

**Figure 2:**
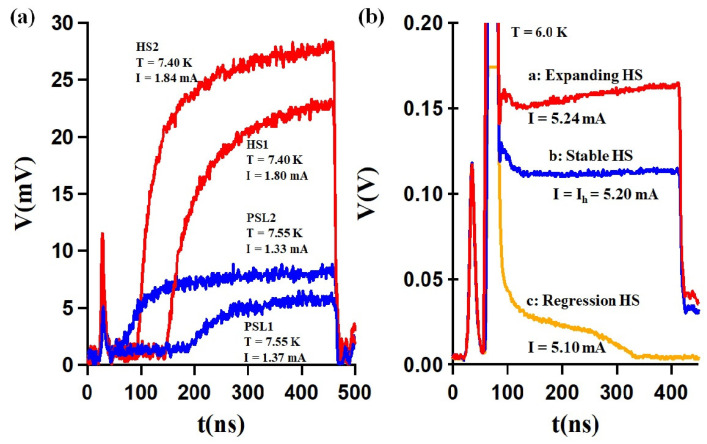
(**a**) Voltage responses of the two distinct dissipative regimes of the 80 nm thick NbTi bridges. At T = 7.4 K < $T^{*}$ the hot spot (HS) is created (HS1 and HS2 correspond to applied currents $I_{HS2}$> $I_{HS1}$). At T = 7.55 K > $T^{*}$ the phase-slip line (PSL) is nucleated (PSL1 and PSL2 correspond to applied currents $I_{PSL2}$> $I_{PSL1}$). (**b**) The response to two-step current pulses at T=6 K: one causing formation of the HS, while the second supports the HS in the expansion (I > $I_{h}$), stable (I = $I_{h}$), or regression modes (I < $I_{h}$).

In a superconducting nanowire, with width comparable to ξ, biased with current slightly below its critical current, any thermal excitation (such as one caused by photon absorption) will cause the transition to the normal state. A localized excitation such as the impact of a photon will locally induce a normal zone and oblige the current to flow around it, which causes current density to exceed the critical value $J_{c}$ around the initial normal zone and so cascade the entire width of the sample into a hot spot. The measured peak voltage is then used as a marker for the detection of the induced resistive state. A similar formation of a hot spot can be obtained by using an electrical current pulse. In this case, a voltage appears after a certain delay time $t_{d}$, after superconductivity locally collapses and a non-equilibrium resistive zone emerges. This zone is brought to a temperature higher than the critical temperature, where quasi-particles are generated. The traces in Figure 2a show the formation of such a hot spot (HS) and the nucleation of a phase-slip line (PSL) in our experiment, respectively, away from $T_{c}$ and in the vicinity of $T_{c}$, for the 80 nm thick NbTi bridge. The formation of an HS is accompanied by a monotonically increasing voltage after $t_{d}$ while the PSL exhibits step-like behavior with a voltage attaining a plateau at times beyond $t_{d}$. To discriminate between the two dissipative modes and accurately measure the hot spot current, we utilized a method involving two superposed pulses. This technique enabled us to move beyond the traditional reliance on the classic hysteresis associated with retrapping currents usingthe I-V characteristics. Instead, we defined a “hot spot current”, analogous to the retrapping current, offering a more precise analysis of the system’s behavior. The first pulse with a duration of 50 ns and a current amplitude set slightly above $I_{c}$ aimed to generate a hot spot. The second pulse with variable amplitude was used to stabilize the HS and avoid its expansion. Figure 2b shows the voltage response to two superposed pulses, the HS created by the first pulse followed by a linear voltage increase for the rest of the remaining time for a current larger than $I_{h}$. By lowering the current amplitude during the second pulse, one can produce a steady hot spot and thus define a threshold hot spot current. The trace *b* of Figure 2b presents a stable voltage, which marks the value of the threshold current for a stable hot spot, yielding a constant voltage response. However, if one further reduces the amplitude of the second current pulse, one will reach the hot spot regression regime (line *c* of Figure 2b). Conversely, larger amplitude of the second pulse may support the expansion of the hot spot (line *a* of Figure 2b), but the range of current amplitudes for such expansion is limited.

**Figure 3:**
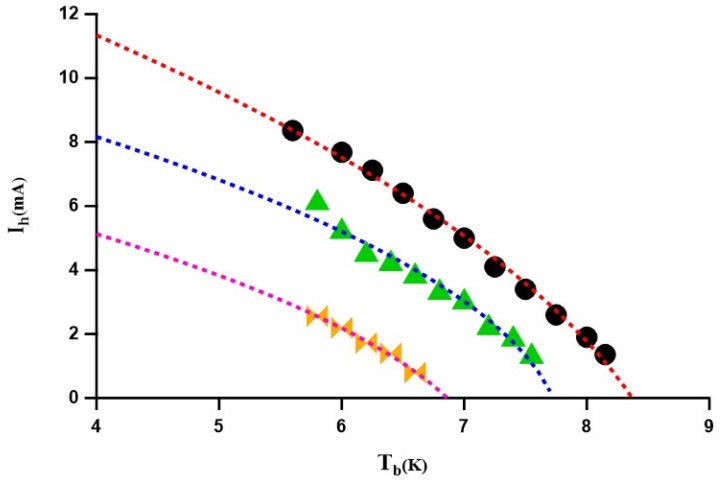
The threshold hot spot current, measured using two-step current pulses, versus the substrate temperature, for different thickness of the NbTi bridge (30, 50 and 80 nm). The experimental data close to $T_{c}$ are well fitted with the (1 − T/$T_{c}$${)}^{0.5}$ dependence, in accordance with theoretical predictions in Ref. [41].

By systematically repeating the above procedure at different temperatures and monitoring the dissipative processes as discussed in Figure 2, we measured and mapped out the temperature dependence of the threshold hot spot current for three NbTi filaments of different thickness, as presented in Figure 3. The obtained values are in excellent functional agreement with the model proposed in Ref. [41], with the energy dissipated as $R_{N}$$I_{h}^{2}\propto$(T − $T_{c}$), i.e., $I_{h}$(T)∝(T − $T_{c}$${)}^{1/2}$.

## 4. Analysis of Delay Time and Gap Relaxation Time

The delay time $t_{d}$ is regarded as the time needed to locally destroy the superconductivity upon external current action. The time-dependent Ginzburg–Landau theory stipulates that $t_{d}$ depends on the normalized order parameter *f*, the ratio between the applied and the critical current, and a prefactor $\tau_{\Delta }$, as

(1) $$t_{d}\left(I/I_{c}\right)=\tau_{\Delta }\int_{0}^{1}\frac{2f^{4}df}{\frac{4}{27}{\left(\frac{I}{I_{c}}\right)}^{2}-f^{4}+f^{6}}.$$

Previous studies showed that delay time is related to the energy gap relaxation time [42]. When Cooper pairs are dissociated, quasi-particles are excited to high energy levels; they relax to the bottom of the energy band where the recombination process takes place and the heat is evacuated to the substrate. $\tau_{\Delta }$ is identified as the gap relaxation time of the filament.

**Figure 4:**
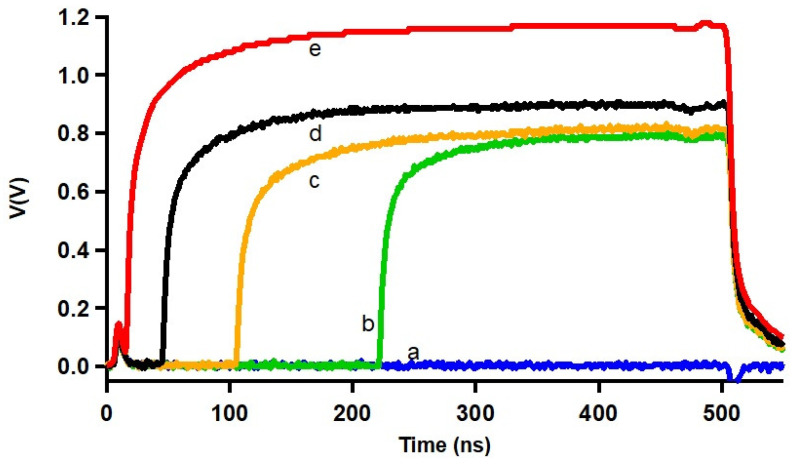
Temporal evolution of the voltage induced by a single-step current pulse in a 80 nm thick NbTi bridge at T = 5.75 K. Voltage onsets after a certain delay time $t_{d}$, with $t_{d}$ reduced with increasing applied current $I/I_{c}=$ 1, 1.02, 1.07, 1.12, and 1.25 (lines labeled **a**–**e**, respectively).

Figure 4 reveals that the delay time $t_{d}$ decreases as the applied single-pulse current increases, which is intuitively expected. This reduction in delay time is correlated with the phonon escape time, reflecting the cooling dynamics of the superconducting film. The evolution of energy dissipation after the creation of the non-equilibrium hot spot also has $\tau_{\Delta }$ as a characteristic time. It implicates the contribution of phonons and electrons, in contrast to the evacuation of the heat into the substrate where only phonons are involved for the period of time $\tau_{esc}$. We thus have $\frac{\int c_{e}dT+\int c_{p}dT}{\tau_{\Delta }}=\frac{\int c_{p}dT}{\tau_{esc}}$, where $c_{e}$ and $c_{p}$ are the specific heat of the electrons and phonons, respectively. The heat escape time can be deduced from the previous equation as $\tau_{esc}=\tau_{\Delta }c_{p}/(c_{p}+c_{e}$). The specific heat values $c_{e}$ and $c_{p}$ in our case can be considered close to the Nb values [43]. Taking the same ($c_{e}$+$c_{p}$)/$c_{p}≃3$, as in Nb, we obtain $\tau_{esc}≃\tau_{\Delta }/3$.

### 4.1. Thickness Dependence of Gap Relaxation Time

The interface between the superconducting film and the substrate influences the nature of the non-equilibrium dissipative state that forms within the film. This boundary interface significantly impacts phonon behavior and, as a result, affects the thermal conductivity in addition to its role in transport measurements. On that note, while earlier study of nanowires illustrated the drop in the thermal conductivity at a rough interface, that is not observed for a smooth interface [44]. The theoretical model developed by Rothwarf and Taylor [45], showed that phonons with energies larger than twice the superconducting gap had an influence on the generated quasi-particles. In such a case, multiple subsequent pair destruction and recombination processes cause the increase in the quasi-particles’ population. Consequently, the lifetime of the quasi-particle becomes longer with increasing the acoustic mismatch between the film and the substrate, which slows the phonon escape process from the film towards the substrate. The recombination time of the quasi-particles depends on the thickness of the film (*d*) and the pair-breaking mean free path ($l_{ph}$). If $d\geq l_{ph}$, the effective time is susceptible to the film thickness; conversely, for $d\leq l_{ph}$ it is independent of the thickness. In case of single-photon detection it is therefore of practical importance to improve the acoustic mismatch between the film and the substrate to reduce the heating effect [46].

The heat generated by a current exceeding the critical value increases the population of the phonons in the localized zone and scales with the thickness. Different combination processes contribute to the energy relaxation rate, notably the electron–phonon scattering, the recombination phenomena, and the electron impurity scattering. The first process enables the modification of the quasi-particle energy through interaction with phonons. In contrast, the second process involves the condensation of quasi-particles into Cooper pairs, accompanied by phonon emission. The acoustic mismatch between the two lattices plays a role in reducing the transmission factor η, but such variation in the transmission coefficient may be omitted in the present analysis since all films of different thickness were evaporated using the same technique and on the same substrate. However, the dominant mechanism is the one with the largestphonon population, where the phonon–phonon interactions escalate and phonons impede each other, causing the phonon transport to became diffusive rather than ballistic. That slows down the evacuation of the heat from the film to the substrate and causes elongation of the heat escape time from the film to the substrate.

**Figure 5:**
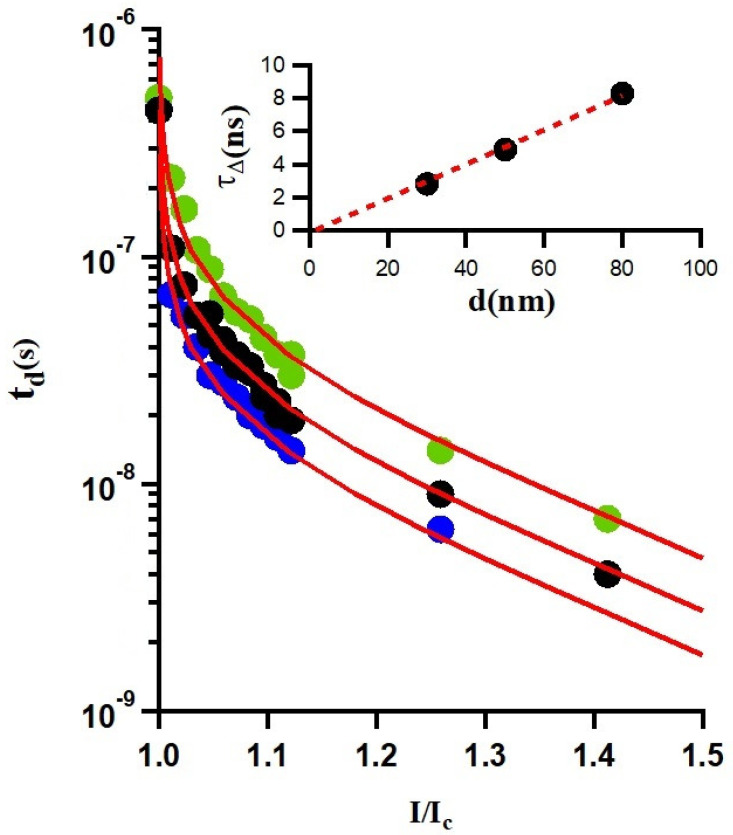
Delay times (in log scale) as a function of the applied reduced current I/$I_{c}$, at T =3.6K (extracted from Figure 4). The red curve is the TDGL functional behavior given by Equation (1), yielding determination of $\tau_{\Delta }$= (2.8 ± 0.2) ns, (4.9 ± 0.2) ns, and (8.3 ± 0.3) ns, for three considered thicknesses of the sample. The inset shows the corresponding escape time $\tau_{esc}\approx \tau_{\Delta }/3$ (see discussion in the text).

Within the scope of the heat transfer between the superconducting material and the substrate, one can conveniently rely on the acoustic mismatch model [46]. For simplicity, we assume that the superconducting films and their substrates could be approximated as isotropic solids. As mentioned earlier, the heat escape time depends on the phonon mean free path $l_{ph}$, the film thickness, and the transmission coefficient η. The mean escape time can then be approximated in case of $d\geq l_{ph}$ by:

(2) $$\tau_{esc}=4d/\left(u\eta \right)\propto d,$$

where *u* is the velocity of sound in the film. The $\tau_{esc}$ depends solely on thickness in the present investigation. The estimate of the heat escape time from the TDGL approach is in good agreement with the acoustic mismatch model, as the experimental data shown in (the inset of) Figure 5 exhibit a linear variation in the gap relaxation time with the film thickness $\tau_{\Delta }/4d\approx 26$ ps/nm ($\tau_{esc}/4d\approx 8.6$ ps/nm). Based on the classical isotropic acoustic mismatch model, the values of $\tau_{esc}/4d$ were estimated from different measurement techniques and reported for some materials and substrates in the past, but these data are rather scarce. For example, Kardakova et al. reported an electron–phonon relaxation time of $\tau_{e-ph}=2$ ns for 80 nm thick TiN film, and the heat escape time of the order of few ns [42]. A comprehensive analysis of the electron–phonon interaction and heat escape times is beyond the scope of this paper, as these factors are highly dependent on the properties of the films, such as their transition temperature, resistivity, and critical current density, all of which are influenced by the film quality.

### 4.2. Effect of Substrate on Characteristic Time Scales

**Figure 6:**
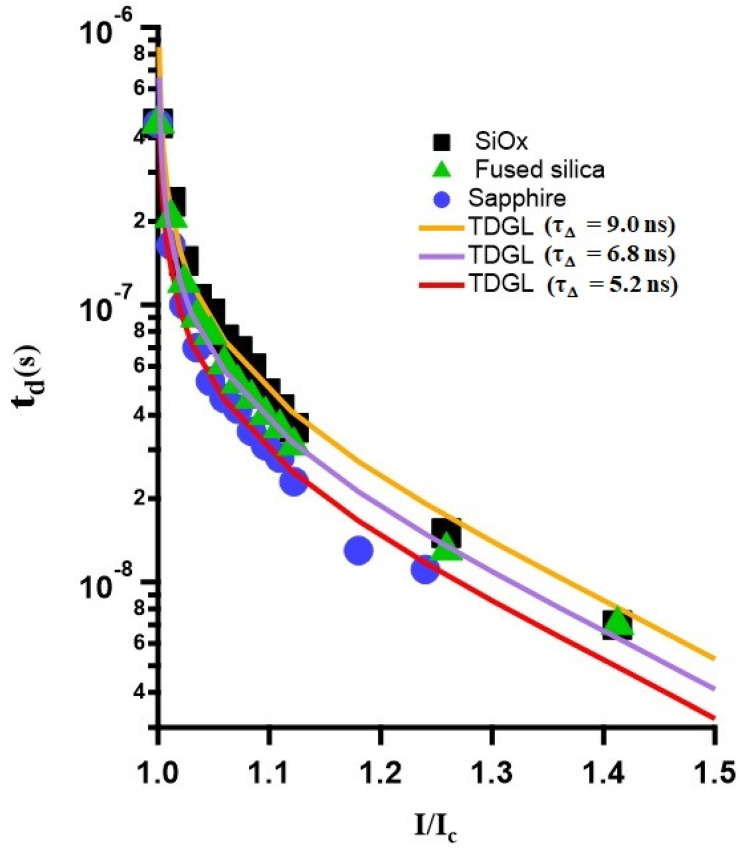
Delay time versus applied current characteristics of a 50 nm thick and 5 μm wide NbTi filament deposited on Si$O_{x}$ (black squares), fused silica (green triangles), and sapphire (blue circles), at temperature T = 5.75 K. The solid lines are the fitting curves using Equation (1), with a prefactor $\tau_{\Delta }$ = (5.2 ± 0.2) ns, (6.8 ± 0.2) ns, and (9.0 ± 0.2) ns for sapphire, fused silica, and Si$O_{x}$ substrates, respectively.

Having measured the thickness dependence of the characteristic dissipative times in a NbTi superconducting filament on a given substrate, we extend our investigation to filaments of the same thickness (in this case 50 nm) deposited on three different substrates. The delay times measured from NbTi filaments in response to a single current pulse on sapphire, fused silica, and Si$O_{x}$ substrates are plotted against their corresponding current values in Figure 6. These data are fitted using the TDGL model using Equation (1), allowing us to extract the characteristic gap relaxation time $\tau_{\Delta }$. The thereby obtained gap relaxation times are $\tau_{\Delta }$ = 5.2 ns, 6.8 ns, and 9 ns for the NbTi filaments on Si$O_{x}$, fused silica, and sapphire, respectively.

The temperature reached at the core of the dissipative zone exceeds the substrate temperature [40]. As a result, heat is generated in a normal zone positioned between two superconducting sinks and is transferred to adjacent areas via phonons and quasi-particles, which leads to the expansion of the HS region. At the same time, heat is also conducted to the substrate, mainly through phonon interactions. As previously discussed, the non-equilibrium system can be divided into two subsystems, quasi-particles and phonons. The Rothwarf–Taylor model was used to describe the time evolution and the dynamics of these two subsystems. The energy dissipation in the non-equilibrium zone occurs with characteristic time $\tau_{\Delta }$. This mechanism of dissipation evolves both electron–electron and electron–phonon interactions. However, this generated heat is evacuated towards the substrate and is assured by the phonon–phonon interactions. This phenomenon is thus in tight dependence with the quality of the interface between the superconducting filament and the substrate.

In our case, the gap relaxation time for the film on sapphire was found to be shorter compared to films on fused silica and Si$O_{x}$ substrates. The sapphire substrate, having a single crystal structure, contrasts with the amorphous structures of the fused silica and Si$O_{x}$ substrates. The morphology of the substrate at the interface significantly influences the phonons responsible for dissipating heat into the substrate sink. In the case of sapphire, diffusive phonons primarily facilitate heat transfer, whereas for fused silica and Si$O_{x}$, ballistic phonons dominate.

Exploring the heat transfer between the superconducting material and the substrate involves using the acoustic mismatch model [45]. Ballistic phonons, characterized by coherent and uninterrupted motion, and diffusive phonons, which exhibit more random and scattered movement, both affect the efficiency of heat dissipation at the interface. Understanding these phonon characteristics is crucial for optimizing heat dissipation strategies in superconducting systems with various (substrate) interfaces, especially in modern circuit designs. When evaluating heat transfer between a superconducting material and a substrate, thermal conductance (*G*) is a key factor to consider. Thermal conductance is given by $G=A\frac{k_{s}\cdot k_{sc}}{k_{s}+k_{sc}}$, where $k_{s}$ is the thermal conductivity of the substrate, $k_{sc}$ is the thermal conductivity of the superconducting material, and A is the cross-sectional area of the interface. This equation accounts for the combined thermal conductivities at the interface, providing insights into heat transfer efficiency.

At temperatures significantly below the superconducting transition temperature ($T_{c}$), the thermal conductivity of the superconducting film is predominantly influenced by phonons, making the interface quality and phonon mean free path ($l_{ph}$) critical factors. For ballistic phonons, their large mean free path indicates less frequent collisions and more coherent motion. This coherent motion impacts heat transport across the interface, affecting overall heat transfer efficiency.

In cryogenic environments, sapphire’s higher phonon transmission typically results in more favorable thermal boundary conductance. For superconducting films used in high-performance electronics or quantum computing, a substrate with higher thermal conductivity like sapphire aids in better heat removal.

For layered structures or devices where thermal management is critical, understanding and optimizing interfacial thermal conductance is essential. Techniques like Time-Domain Thermoreflectance (TDTR) can measure and optimize these properties. Sapphire, with better thermal conductivity and longer mean free paths compared to fused silica and Si$O_{x}$, has a thermal conductance relationship $G_{sapphire}$ < $G_{fusedsilica}$ < $G_{SiOx}$. This aligns with our measured heat escape times, where sapphire shows faster escape times compared to fused silica, and Si$O_{x}$ exhibits the slowest one. This order of escape times, $\tau_{sapphire}<\tau_{fusedsilica}<\tau_{SiOx}$, summarizes the distinct thermal dynamics of these substrates.

Assuming that superconducting films and substrates are isotropic solids and the films are sufficiently thick, both the gap relaxation time and heat escape time will depend on the film thickness, as specified by Equation (2). Using the same filament thickness in our analysis of the effect of the substrate, the transmission coefficient η remains as the only variable. Thereby estimated values of uη for NbTi on sapphire, fused silica, and Si$O_{x}$ are 12.82, 9.80, and 7.41 m/s, respectively. This in turn yields the thickness dependence of the characteristic heat escape time as $\tau_{esc}=312$ ps per nm, 408 ps per nm, and 540 ps per nm, for NbTi on sapphire, fused silica, and Si$O_{x}$, respectively. This information is of high practical value towards the optimized design of superconducting sensing devices based on heat absorption and general optimization of superconducting electronics where excess heat has detrimental effects that need be controlled if not removed.

## 5. Conclusions

To extract the characteristic time scales of gap and heat relaxation in thin superconducting filaments, we conducted pulsed-current transport measurements on 2D NbTi superconducting micro-bridges of different thickness and on different substrates, to investigate their temporal response to overcritical current, and their behavior in the resistive state. For that goal, we first established the threshold current for a stable resistive state, and mapped out its dependence on temperature. Then, for a given temperature, we detected the temporal delay of the suppression of superconductivity, as a function of applied current. After corroborating the data with the time-dependent Ginzburg–Landau (TDGL) model, we determined the gap relaxation and heat escape time, which display a linear correlation with film thickness, aligning with predictions from the acoustic mismatch model. Specifically, thin NbTi filaments on sapphire exhibited a fast gap relaxation time of 104 ps per nm of thickness, suggesting suitability of such nanostructures for advanced superconducting electronics. We extended our investigation into gap relaxation time across various substrates (sapphire, fused silica, and SiO*x*), to highlight the crucial influence of the interface with the substrate. We report slower heat escape times for SiO*x* and fused silica substrates compared to sapphire, which can be attributed to different phonon transport mechanisms and an amorphous interface. These results and insights underscore the importance of substrate selection and interface quality in optimizing the performance of superconducting detectors and electronic devices, which is pivotal, e.g., for advancing superconductor-based single-photon detectors and bolometers.

## Data Availability

The original contributions presented in the study are included in the article. Further inquiries can be directed to the corresponding author.
